# GWAS and WGCNA analysis uncover candidate genes associated with drought in *Brassica juncea* L.

**DOI:** 10.3389/fpls.2025.1551804

**Published:** 2025-04-04

**Authors:** Yusong Zhang, Xiaoyan Yuan, Yunyun Zhang, Yanqing Luo, Kaiqin Zhao, Feng Zu, Zhengshu Tian, Jinfeng Li, Lifan Zhang, Xiaoying He, Jinxiang Gao, Minglian Fu, Genze Li, Feihu Liu

**Affiliations:** ^1^ Industrial Crops Research Institute, Yunnan Academy of Agricultural Sciences, Kunming, China; ^2^ Yunnan Key Laboratory of Genetic Improvement of Herbal Oil Crops, Kunming, China; ^3^ College of Agriculture, Yunnan University, Kunming, China

**Keywords:** *Brassica juncea* L., drought, GWAS, WGCNA, selective sweep analysis, genes

## Abstract

Drought poses a major challenge to crop growth and yield, and exploring the drought tolerance of crops is an effective and economical approach to mitigating the effects of drought. To screen drought-tolerant germplasm resources and key functional genes related to drought tolerance in *Brassica juncea* L.(193 accessions), three treatments were applied at the germination and seedling stages:control(CK), moderate drought stress (M), and severe drought stress (S). Drought tolerance identification, GWAS, and RNA-Seq analysis of these materials under different treatments showed that drought stress significantly reduced the germination rate, aboveground and underground fresh weight at the seedling stage, harvest index at maturity, and expanded the root/shoot ratio. From the 193 materials, 24 drought-tolerant, 139 drought-tolerant medium, and 30 drought-sensitive materials were identified. The 77 SNPs identified by GWAS were associated with the relative germination rate at the germination stage, and the fresh weight of the aboveground and underground parts at the seedling stage, which could be integrated into 27 QTLs. WGCNA identified 15, 0, and 5 modules significantly related to drought tolerance in the aboveground and underground parts at the germination and seedling stages, respectively. By correlating the significant GWAS SNPs with the significant WGCNA modules, a total of 11 genes related to drought tolerance under moderate and severe drought stress were identified. These genes were involved in the regulation of auxin-responsive protein (*SAUR*), *LEA* protein, glucosidase, *AP2/ERF*, *WRKY* and *GATA* transcription factors, *FLZ* zinc finger domain, *PRP*, and *b561* proteins. Among them, the *BjuB035910* gene was detected in the underground parts of the seedling and germination stages under moderate drought stress. GWAS and selective sweep analysis jointly identified the 23.955-24.089 Mb region of chromosome B06, where four genes (*BjuB022264, BjuB022292, BjuB022282*, and *BjuB022235*) were located, as confirmed by WGCNA analysis. A total of 125 SNPs with high linkage disequilibrium were found in this region, and 12 haplotypes were detected, with Hap1 being present exclusively in drought-tolerant materials and Hap3-Hap12 distributed in drought-sensitive materials. These findings provide new insights into the drought tolerance mechanisms of *B. juncea* and will contribute to the breeding of drought-tolerant rapeseed varieties.

## 1 Introduction

Abiotic stresses, such as drought, are among the main factors contributing to the reduction of global crop yields, resulting in the loss of more than 50% of the world’s major crop yields each year (Kopecká et al., 2023). Over the past decade, drought has caused a total of $30 billion in global crop production losses, with tropical regions suffering from more than 20 million tons of crop yield loss almost annually (Verma and Deepti, 2016; Zhang H. et al., 2022). This phenomenon is expected to worsen as global climate change progresses, with 5 billion people living in dryland areas and a 50% reduction in freshwater availability by 2050 (Gupta et al., 2020). Therefore, research on plant drought tolerance and the cultivation of drought-tolerant crops are crucial to ensuring global food security.

Rapeseed is the largest oil crop in China and a crucial source of vegetable oils and proteins. Rapeseed oil accounts for more than 40% of China’s total self-produced edible vegetable oil. With the improvement in living standards, the demand for edible oil is expected to continue to rise (Fu et al., 2016; Fong and Wang, 2024). Currently, the most widely cultivated rapeseed variety in China is *Brassica napus*, which requires a significant amount of water throughout its growing season. This variety is also poorly adapted to drought conditions and is prone to drought damage (Pang and Wang, 2023). Moreover, winter rapeseed, which accounts for about 90% of the total rapeseed area in China, is mainly grown in the Yangtze River Basin, the Yunnan-Guizhou Plateau, and other production regions (Hu et al., 2017). Winter rapeseed production in China often faces challenges such as uneven seedling emergence and weak seedlings, which can adversely affect yields, particularly due to autumn droughts during the seedling stage (Su et al., 2014; Xiong et al., 2015; Hou et al., 2018; Huang et al., 2019a, 2019b). Breeding and planting drought-tolerant rapeseed varieties are key measures to address this production issue. *B. juncea*, an important rapeseed type in China, has been widely cultivated in arid areas of central and western China, as well as in India, Pakistan, and other countries. It is known for its drought tolerance, resistance to soil infertility, and resistance to diseases such as black shank and split angle, making it suitable for planting in arid and semi-arid regions (Yuan et al., 2023). Yunnan, a major area for high-quality crude oil rapeseed production in China, is located on a low-latitude plateau with complex landforms, diverse climates, and abundant mustard rapeseed germplasm resources (Zhang et al., 2012). Furthermore, Yunnan experiences distinct dry and wet seasons, with seasonal droughts being common in winter and spring. As a result, the drought tolerance and soil infertility resistance of many *B. juncea* varieties in Yunnan are particularly notable (Yuan et al., 2023). Therefore, systematically studying the mechanisms of drought tolerance in Yunnan *B. juncea* and screening drought-tolerant germplasm resources will provide important reference information for improving drought tolerance traits in rapeseed.

Currently, research on drought tolerance in rapeseed mainly focuses on *B. napus*, particularly during the germination stage (Yuan et al., 2020a, 2023). It has been found that drought tolerance in *B. napus* is a complex quantitative trait controlled by multiple genes (Meng et al., 2021). Several drought-tolerance-related genes have been identified through QTL mapping, GWAS, and RNA-Seq. For example, by applying PEG-6000 stress at the germination stage of *B. napus*, 43 significantly associated SNP markers and 37 candidate genes were identified using seed germination rate and germination index via GWAS (Tan et al., 2017). After treating 228 *B. napus* plants with PEG stress, 314 significantly associated SNPs and 85 candidate genes were identified through GWAS (Khanzada et al., 2020). Using transcriptome sequencing, 169 differentially expressed genes were screened in drought-tolerant and drought-sensitive materials of *B. napus* (Wang et al., 2017). By performing transcriptome sequencing on the roots and leaves of *B. napus* under both normal irrigation and drought stress, 6018 and 5377 differentially expressed genes were identified in the roots and leaves, respectively (Liu et al., 2015). Through the creation of introgression lines (*ILs*) by hybridizing *B. carinata* with *B. juncea*, 29 additive QTLs for drought tolerance were identified using IL linkage maps, 17 of which originated from *B. carinata* (Limbalkar et al., 2023). Despite these advancements, research on the drought tolerance mechanisms of *B. juncea* remains limited. Key aspects such as the utilization of superior traits, screening for drought-tolerant germplasm, and the identification of key drought tolerance genes have not been thoroughly explored (Liu et al., 2010; Wang et al., 2020a; Huang et al., 2022). Therefore, further research on gene mining and the drought tolerance mechanisms of *B. juncea* is of great significance for improving the drought tolerance of rapeseed.

Drought tolerance during the seedling stage is crucial for the growth and yield of rapeseed, particularly in regions like China where climate and environmental factors play a significant role. Improving drought tolerance at this stage is key to stabilizing rapeseed yields. In this study, 193 *B. juncea* germplasm resources from Yunnan Province were used to explore the genes associated with drought tolerance during germination and the seedling stages through GWAS and WGCNA. The findings lay a foundation for the breeding of drought-tolerant rapeseed varieties.

## 2 Materials and methods

### 2.1 Plant materials

A total of 329 phenotypically rich primary germplasms were selected from 560 *B. juncea* resources in the germplasm resource bank of the Yunnan Academy of Agricultural Sciences. To evaluate the population genetic polymorphism of these primary germplasms, 36 pairs of SSR markers, evenly distributed across the *B. juncea* genome, were used. Based on the genetic clustering information of these SSR markers, 193 core germplasms were selected as research materials (**Supplementary Table S1**). Among them, 161 were Yunnan landraces and bred varieties, while 32 came from other provinces and abroad. Specifically, 15 were from Guizhou, 2 from Hubei, 5 from Sichuan, 1 each from Shanxi, Chongqing, and Xinjiang, and 6 were from India, with 1 from Poland.

### 2.2 Drought stress treatment at germination stage

Three drought treatments were set: control (CK) (deionized water); moderate drought (M) (19% PEG-6000); severe drought (S) (22% PEG-6000) (Yuan et al., 2012). Each treatment was repeated three times, with 100 seeds per replicate. The seeds were disinfected with 1% sodium hypochlorite for 15 minutes, then rinsed with water six times. Afterward, the seeds were placed in a 12 cm diameter, 2.5 cm high circular Petri dish with double-layer filter paper as the bedding for germination. Ten milliliters of the stress solution or deionized water was added, and the seeds were cultured at a constant temperature of 20°C. The test was repeated three times in October 2019 (E1), October 2020 (E2), and October 2021 (E3).

### 2.3 Drought stress treatment at seedling stage

Seedling drought stress experiments were conducted in 2019 (E1), 2020 (E2), and 2021 (E3) in a glass greenhouse. The soil in each pot (68cm×38cm×40cm) was uniform. After sowing, seedlings were watered to ensure germination, and 8 plants with consistent growth were left in each pot. There were 3 pots for each treatment, totaling 193 materials. Seedlings were allowed to grow normally for 10 to 15 days (about 3 to 4 true leaves), after which drought stress treatments were applied for 35 days. Three treatments were applied at the seedling stage: CK (normal irrigation after seedling emergence), M (irrigation amount was 2/3 of theCK), and S (irrigation amount was 1/2 of theCK), with each treatment repeated 3 times. After the drought stress period, all treatments were watered normally until the seeds matured.

### 2.4 Phenotypic data statistics

The number of germinated seeds for each material was investigated on day 7 after sowing during the germination stage. The SPAD-502 chlorophyll meter was used to measure the chlorophyll content of the 3rd and 4th leaves from top to bottom of each material after drought stress at the seedling stage. For each treatment, the fresh weights of the shoots and roots were measured from five plants, and the grains of ten plants were dried and weighed after maturity. The relative germination rate (RG), relative chlorophyll content (RC), relative aboveground fresh weight (RAW), and relative root fresh weight (RUW) were calculated using Equation 1. The drought tolerance index (DI) was calculated using Equation 2, and the D value for comprehensive drought tolerance evaluation was calculated using Equations 3–5.


(1) trait relative value(TR)=Xd/Xc

(2) $$\text{Drought tolerance index}\left(DI\right)=\left(\frac{YD}{YP}\right)\times \bar{YD}$$

In Equation 1, *Xd* and *Xc* are the measured values of the traits under drought stress and control, respectively. In Equation 2, *YD* is the yield under drought stress; *YP* is the yield under normal irrigation conditions, and *Y* $\overset{—}{D}$ is the average of all YDs.


(3) $$\text{Factor weighted coefficient}\omega_{i}=P_{i}÷\sum_{i=1}^{n}P_{i},i=1,2,3,\ldots ,n.$$

In Equation 3, *P_i_* is the contribution rate of the ith composite indicator, which indicates the importance of the ith indicator among all indicators.


(4) $$\text{The value of the membership function for each metric }\mu \left(X_{i}\right)=\frac{X_{i}-X_{imin}}{X_{iMax}-X_{imin}},i=1,2,3,\ldots ,n.$$

In Equation 4, *X_i_, X_imin_*, and *X_imax_* represent the minimum and maximum values of the ith index and the ith composite index, respectively.

According to the factor weighted coefficient *(ω_i_)* and the membership function value *μ (X_i_)*, the *D* value of drought tolerance was calculated.


(5) $$D=\sum_{i=1}^{n}\left[\mu \left(X_{i}\right)\times \omega_{i}\right],i=1,2,3,\ldots ,n$$

### 2.5 Sequencing quality control and genome-wide association analysis

A total of 193 genomic DNA samples were extracted from young leaves of *B. juncea* to construct a DNA library, which was then sequenced using the Illumina Nova 6000 platform. Adapter sequences and low-quality reads (those with a Qphred score ≤ 10, where base calls account for more than 20% of the read length, or with ≥5% unrecognized nucleotides) were removed from the raw data. The remaining clean data were then reassembled to eliminate redundancy. The clean data were aligned to the *B. juncea* reference genome (http://39.100.233.196:82/download_genome/Brassica_Genome_data/Braju_tum_V1.5/) using BWA v0.7.13. Single nucleotide polymorphisms (SNPs) and insertions and deletions (InDels) were detected using GATK software (Wei et al., 2021). The variant sites were filtered, and association analysis was conducted using the EMMAX model. The significance threshold for tested traits was determined using the formula P = 0.05/n (where n is the number of SNPs). Related genes within 100 kb of significantly correlated SNPs were identified. QQ plots and Manhattan plots were generated using the CMplot package (García-Fernández et al., 2021).

### 2.6 Selective sweep analysis

Based on the results of drought tolerance identification, 10 drought-tolerant and 10 drought-sensitive materials were selected under moderate drought stress at the germination stage, 15 drought-tolerant and 10 drought-sensitive materials under severe drought stress, and 2 drought-tolerant and 2 drought-sensitive materials at the seedling stage for selective sweep analysis. The population differentiation coefficient (Fst) of each group was separately calculated using vcftools software along each chromosome with a 100 kb window and a 10 kb sliding step (Zhou et al., 2024). The top 5% of intervals were selected as the selective sweep intervals, and candidate genes within these intervals were identified.

### 2.7 Transcriptome sequencing data analysis

Based on the results of drought tolerance identification, two drought-tolerant and two drought-sensitive materials were selected. RNA was extracted from the leaves and roots at both the germination and seedling stages under CK, M, and S treatments. The RNA was then sequenced using the Illumina Novaseq 6000, and gene expression levels were measured based on the FPKM value. Differentially expressed genes (DEGs) were identified using Cuffdiff software (Shi et al., 2022), with the following criteria: (1) fold change (FC) > 2; (2) False Discovery Rate (FDR) q-value< 0.05. GO and KEGG enrichment analyses of the DEGs were performed using clusterProfiler software (Jiang et al., 2022).

### 2.8 Gene expression network construction and visualization

WGCNA analysis and visualization were performed using the WGCNA package (v1.47) and the ggcor package (Zhang Q. Y. et al., 2022; Chen et al., 2024). The default automatic network builder blockwiseModules was used to construct a co-expression module with a threshold of 9 and a Minimum ModuleSize of 50. Module characteristic genes were then used to calculate the correlation coefficient of the sample. Modular gene co-expression network analysis was performed using Gephi software (https://gephi.org/) (Li et al., 2023).

### 2.9 Correlation analysis of the target area

A heat map of linkage disequilibrium (LD) between SNPs on chromosome B06 was generated and visualized using HaploView software (Dadshani et al., 2021). SNPs or haplotypes associated with drought tolerance were identified using TASSEL 5.0 software (Tong et al., 2024), with a threshold set at −log10(p) ≥ 2.5.

## 3 Results

### 3.1 Phenotypic changes at the germination and seedling stages under drought stress

**Figure 1:**
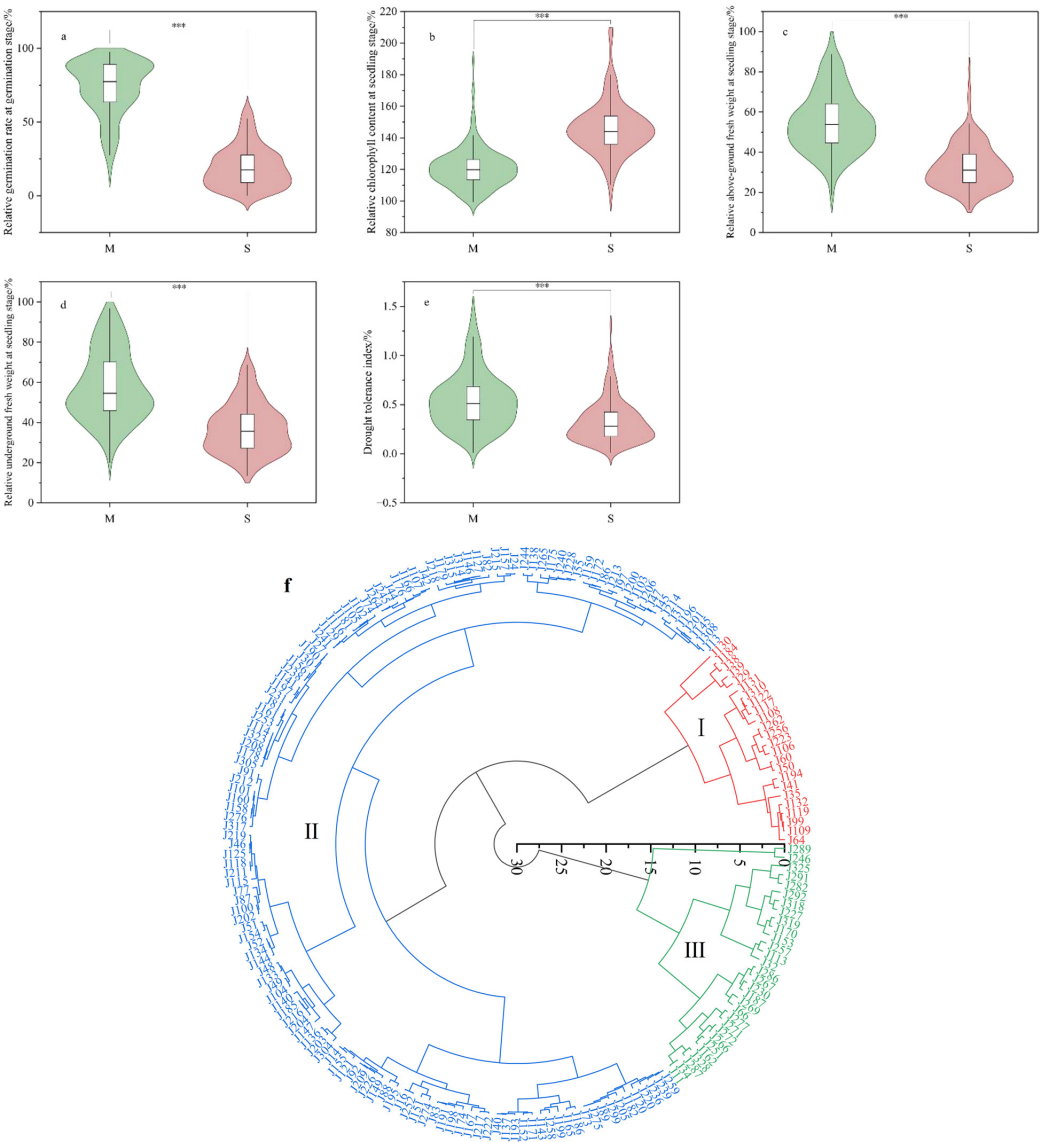
Phenotypic changes under different drought stress treatments. **(a-e)** are the relative values of each trait under moderate stress M (Moderate) and severe stress S (Severe). **(f)** is the cluster analysis diagram of the tested varieties based on the membership function calculated according to the relative values of traits at germination stage and seedling stage.

Rapeseed has relatively weak tolerance to water stress, and drought during the sowing period inevitably leads to difficulties in germination and reduced seedling growth. After simulating moderate (M) and severe (S) drought stress treatments on 193 varieties, growth and development during the germination and seedling stages were affected. The germination rate during the germination stages, fresh weight of the aboveground and underground parts during the seedling stage, and harvest index at maturity were all lower compared to the control. Chlorophyll content was higher than the control, and the greater the drought stress, the larger the changes (**Figures 1a–e**). Under moderate drought treatment, the average relative germination rates of the tested materials in three replicates were 74.22%, 74.19%, and 71.90%, with a coefficient of variation ranging from 25.55% to 27.05% (**Table 1**). Under severe drought treatment, the average relative germination rates were 20.37%, 19.42%, and 18.95%, and the coefficient of variation ranged from 73.31% to 73.69%. These results indicate that the germination rates of materials were relatively stable under moderate drought, whereas the rates varied significantly under severe drought, likely due to differences in drought tolerance among the materials. At the seedling stage, the average relative aboveground fresh weight for the three replicates under moderate drought treatment were 55.35%, 54.40%, and 56.11%, with a coefficient of variation ranging from 27.44% to 29.02%. Under severe drought treatment, the average relative aboveground fresh weight were 33.85%, 31.96%, and 33.22%, with a coefficient of variation range from 33.20% to 36.88%. The mean values for the relative underground fresh weight were 59.50%, 56.87%, and 57.53% under moderate drought treatment, with a coefficient of variation between 29.71% and 32.68%. Under severe drought treatment, the average relative underground fresh weight were 38.25%, 36.11%, and 36.75%, with a coefficient of variation ranging from 32.86% to 37.11%. These findings suggest that drought has a more pronounced impact on the aboveground biomass compared to the underground part at the seedling stage, and it also leads to an increased root-to-shoot ratio. The relative chlorophyll content at the seedling stage was 119.50%, 122.92%, and 120.15% in three replicates under moderate drought treatment, with a coefficient of variation ranging from 10.66% to 11.48%. Under severe drought treatment, the values were 145.99%, 147.54%, and 144.83%, with a coefficient of variation between 11.87% and 12.14%. These results indicate that drought stress increases chlorophyll content and darkens leaf color. The drought tolerance index values under moderate drought treatment were 0.50 and 0.57, with a coefficient of variation ranging from 83.92% to 62.52%. Under severe drought treatment, the values were 0.30 and 0.35, with a coefficient of variation between 82.46% and 106.40%.To evaluate the drought tolerance of the tested materials, we calculated membership function values based on the relative germination rate, relative fresh weight of the aboveground and underground parts at the seedling stage, relative chlorophyll content, and drought tolerance index. The 193 materials were classified into three categories (**Figure 1f**): Category I included 24 drought-tolerant materials, Category II included 139 materials with medium drought tolerance, and Category III included 30 drought-sensitive materials.

**Table 1:** Descriptive statistics of phenotypic traits at germination and seedling stages.

Year	Treats	Traits	Average	Max	Min	Skewness	kurtosis	Coefficient/%
2019	M	RG	74.22 ± 20.08	98.98	16.13	-0.88	-0.07	27.05
		RAW	55.35 ± 15.99	96.55	18.76	0.31	-0.36	28.89
		RUW	59.50 ± 17.68	99.93	20.15	0.32	-0.60	29.71
		RC	119.50 ± 13.33	198.08	95.24	1.95	7.51	11.16
	S	RG	20.37 ± 14.95	63.42	0.00	0.79	0.03	73.38
		RAW	33.85 ± 11.24	82.15	11.33	1.06	1.76	33.20
		RUW	38.25 ± 12.57	75.97	12.51	0.47	-0.18	32.86
		RC	145.99 ± 17.73	217.64	105.24	0.93	2.50	12.14
2020	M	RG	74.19 ± 18.95	101.02	11.33	-0.90	0.28	25.55
		RAW	54.40 ± 15.79	98.99	16.85	0.40	-0.11	29.02
		RUW	56.87 ± 18.58	100.00	17.65	0.41	-0.53	32.68
		RC	122.92 ± 14.11	183.33	93.43	0.89	2.24	11.48
		DI	0.50 ± 0.42	2.30	0.01	1.53	2.58	83.92
	S	RG	19.42 ± 14.23	59.00	0.01	0.83	0.07	73.31
		RAW	31.96 ± 11.79	81.69	9.50	1.06	2.17	36.88
		RUW	36.11 ± 13.40	72.84	10.00	0.51	-0.30	37.11
		RC	147.54 ± 17.51	211.18	93.54	0.65	1.94	11.87
		DI	0.30 ± 0.25	1.79	0.02	2.70	10.73	82.46
2021	M	RG	71.90 ± 18.39	98.79	16.53	-0.94	0.30	25.57
		RAW	56.11 ± 15.40	99.19	15.33	0.40	0.02	27.44
		RUW	57.53 ± 17.34	98.68	22.58	0.35	-0.68	30.14
		RC	120.15 ± 12.81	185.31	29.41	-0.30	10.01	10.66
		DI	0.57 ± 0.36	2.17	0.01	1.02	1.50	62.52
	S	RG	18.95 ± 13.97	62.00	0.00	0.90	0.46	73.69
		RAW	33.22 ± 12.15	82.02	10.89	0.91	1.19	36.58
		RUW	36.75 ± 12.62	69.26	11.34	0.48	-0.21	34.35
		RC	144.83 ± 17.34	208.24	105.76	0.93	1.97	11.97
		DI	0.35 ± 0.38	2.05	0.01	1.71	3.07	106.40

M, Moderate drought; S,Severe drought; RG, Relative germination rate at germination stage; RAW, Relative above-ground fresh weight at seedling stage; RUW, Relative underground fresh weight at seedling stage; RC, Relative chlorophyll content at seedling stage; DI, Drought tolerance index.

### 3.2 Genome-wide association analysis

Based on the phenotypic analysis of 193 materials under moderate and severe drought stress (**Table 1**), the skewness and abundance of the relative germination rate, relative aboveground and underground fresh weights at the seedling stage ranged from -0.94 to 1.06 and -0.68 to 2.17, respectively, which were consistent with a normal distribution. The coefficient of variation ranged from 25.55% to 73.69%, indicating significant variation among the materials. Therefore, relative germination rate, relative aboveground fresh weight, and relative underground fresh weight at the seedling stage were selected for the genome-wide association analysis.

**Figure 2:**
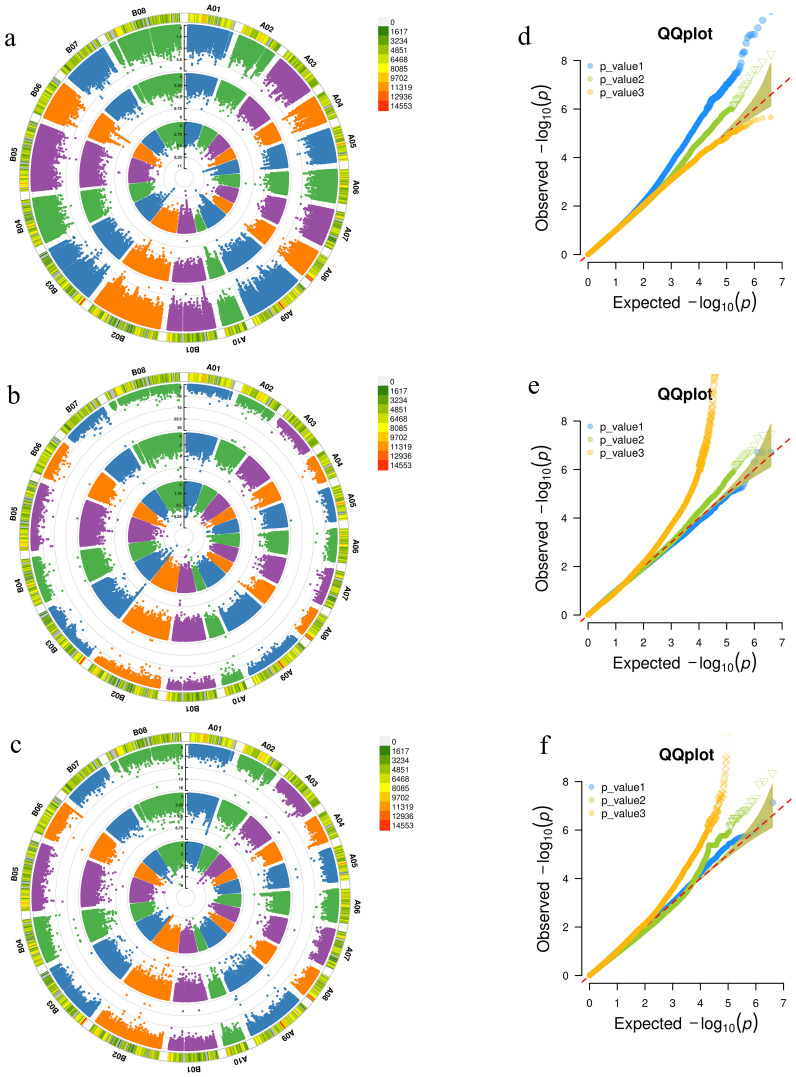
Genome-wide association analysis of Manhattan plot and QQ plot. **(a-c)** are the Manhattan plots of the relative germination rate, the aboveground and underground fresh weight at the seedling stage between 3 years (2019, 2020 and 2021) under moderate drought stress. **(d-f)** are the QQ plots of relative germination rate, aboveground and underground fresh weight at seedling stage in 3 years under moderate drought stress.

The SNP loci significantly associated with the relative germination rate under moderate drought stress at the germination stage in the three environments (E1, E2, E3) were detected by EMMAX (-log10(P)≥4) (**Figures 2a, d**). A total of 2924 SNPs were detected in the E1 environment, 1119 in the E2 environment, and 452 in the E3 environment. Nine SNPs were co-detected in both E1 and E2 environments, located on chromosomes B06 (6), B03 (1), A04 (1), and A07 (2) (**Supplementary Table S2**). According to Liu and Chen et al.’s method (Liu et al., 2016; Chen et al., 2018), these SNPs were integrated into the same QTL if their distance was within 100 kb and the linkage disequilibrium between markers (r2 > 0.35). These nine SNPs were integrated into five QTLs. Taking the 100 kb upstream and downstream regions of SNPs as the candidate interval, the nine SNPs encompassed a total of 978 genes. Under severe drought stress at the germination stage (**Supplementary Figure S1A**), 461 SNPs were detected in the E1 environment, 1161 in the E2 environment, and 978 in the E3 environment. A total of 32 SNPs were detected across the three environments, which could be integrated into one QTL. All of these SNPs were located within 45 kb of chromosome A04 (2036566~2082057), and together, they contained a total of 800 genes.

Under moderate drought stress at the seedling stage, 292 SNPs were detected in the E1 environment, 639 SNPs in the E2 environment, and 2193 SNPs in the E3 environment for relative underground fresh weight. Among them, 2 SNPs were co-detected in both E1 and E3 environments, located on the A02 and B02 chromosomes (**Figures 2b, e**). These two SNPs together contained a total of 175 genes. Under severe drought stress, 1 SNP was co-detected in both E1 and E2 environments, and 5 SNPs were co-detected in both E2 and E3 environments (**Supplementary Figure S1B**). These 6 SNPs contained a total of 672 genes. Under moderate drought stress at the seedling stage, 577 SNPs were detected in the E1 environment, 627 SNPs in the E2 environment, and 2134 SNPs in the E3 environment for relative aboveground fresh weight. Among these, 8 SNPs were co-detected in both E2 and E3 environments, all of which were located on the A06 chromosome and were continuously distributed (**Figures 2c, f**; **Supplementary Table S2**). These SNPs could be integrated into one QTL, and the eight SNPs together contained a total of 130 genes. Under severe drought stress, 597 SNPs were detected in the E1 environment, 2001 SNPs in the E2 environment, and 5024 SNPs in the E3 environment. Among these, 7 SNPs were co-detected in both E1 and E3 environments, and 13 SNPs were co-detected in both E2 and E3 environments (**Supplementary Figure S1C**). These 20 SNPs could be integrated into 12 QTLs, which were distributed on chromosomes A01 (1), A02 (2), A03 (1), B01 (2), B02 (1), B03 (2), B04 (1), B06 (1), and B07 (1). The 20 SNPs together contained a total of 1144 genes.

### 3.3 Selective sweep analysis

The population differentiation coefficients for the drought-tolerant and sensitive groups were calculated using a 100 kb window and a 10 kb step size. The top 5% of the Fst region represented significantly selected regions, with 35 significant selective sweep regions identified, distributed across 18 chromosomes. Regions with strong selection signals were located on chromosomes A01, A03, A04, A05, A09, B01, and B05. The two intervals on the A01 chromosome with the strongest signal were (6,760,001~7,730,000 and 34,140,001~34,620,000), containing 48 and 40 genes, respectively. Combined with the analysis of 41 SNPs significantly associated with the germination stage, identified in the upstream and downstream 100 kb range from GWAS, two regions on chromosome B06 were identified (8,170,361~8,357,420 and 23,686,061~24,177,367), containing 20 and 43 candidate genes, respectively (**Supplementary Table S3**). Among them, 28 have been reported to be associated with drought tolerance in *Arabidopsis thalian*a and other crops, such as ethylene-responsive transcription factors *ERF* and *ABR*, zinc finger transcription factors, *GATA*, cytochrome *b561*, and others. GWAS and selective sweep analysis at the seedling stage identified two regions on chromosome A02 (15,634,807~15,634,893) and chromosome B037(24,327,885~24,737,556), containing 2 and 20 genes, respectively (**Supplementary Table S3**). These genes mainly encode plasma membrane aquaporins (*PIP*), auxin response proteins, disease resistance proteins (*CHS1*), and others.

### 3.4 Transcriptome and WGCNA analysis at germination and seedling stages

**Figure 3:**
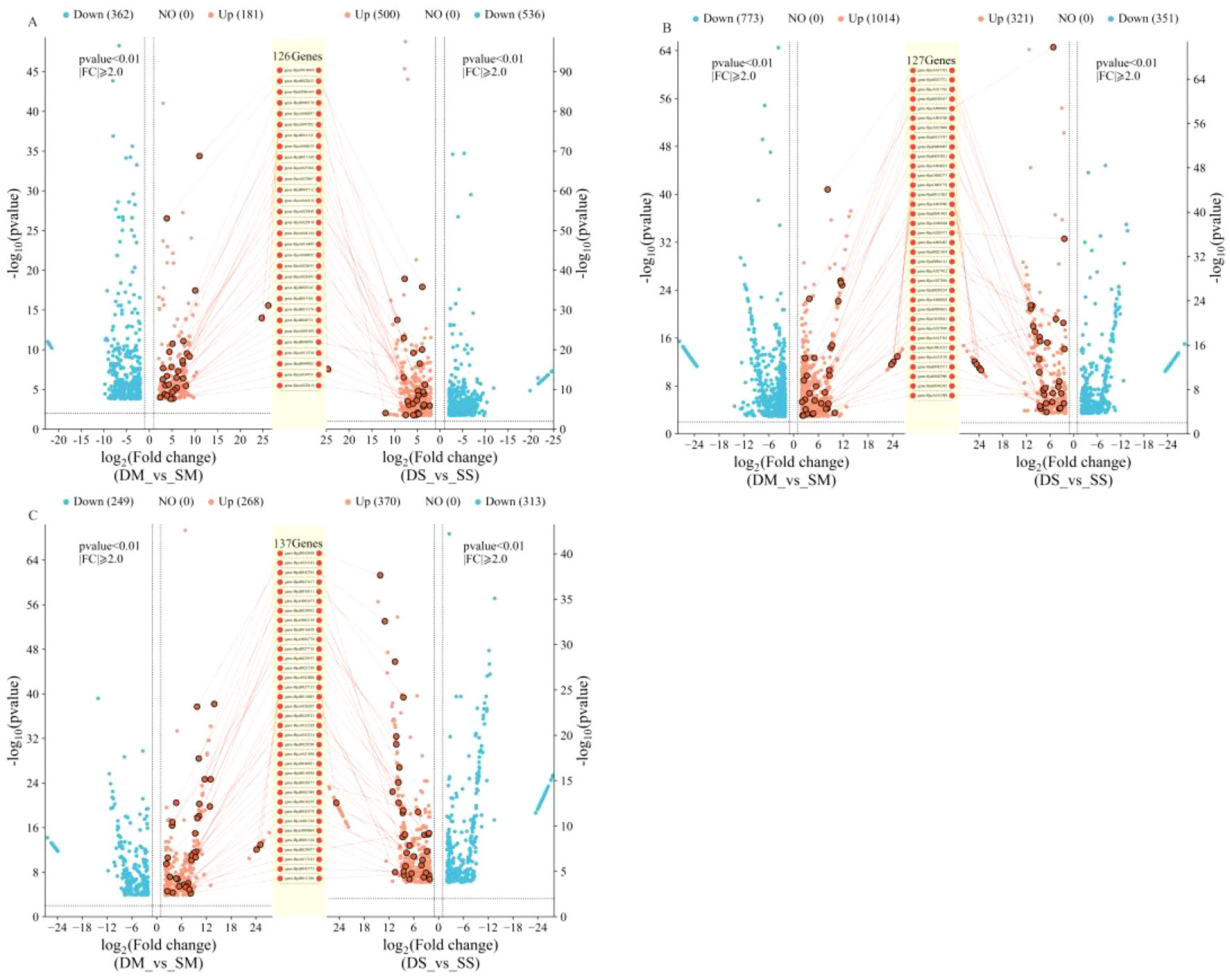
Differentially expressed genes between drought-tolerant and sensitive groups at germination and seedling stages. **(A)** is the germination stage; **(B)** is the underground part of the seedling stage; **(C)** is the aboveground part of the seedling stage. DM, drought-tolerant group under moderate drought stress; SM, sensitive group under moderate drought stress; DS, drought-tolerant group under severe drought stress; SS, Sensitive group under severe drought stress. The names of the genes marked in the figure are co-upregulated genes.

The embryos, leaves, and roots of the drought-tolerant and sensitive groups were sequenced under four conditions: normal irrigation, moderate drought stress, and severe drought stress at both the germination and seedling stages. More than 89% of the effective sequences from each sample were compared to the reference genome (**Supplementary Table S4**), and the sequencing quality was high. The data were filtered using DESeq2 software with parameters set to log_2_FC > 2 and padj< 0.01.Under moderate drought stress at the germination stage, a total of 643 differentially expressed genes (DEGs) were identified in both the drought-tolerant and sensitive groups. Under severe drought stress, 1036 DEGs were identified, with 126 genes co-upregulated under both moderate and severe drought stress (**Figure 3A**). At the seedling stage, 1787 DEGs were identified in the underground parts of both groups under moderate drought stress. Under severe drought stress, 673 DEGs were identified, with 127 genes co-upregulated under both moderate and severe drought stress (**Figure 3B**). In the aboveground parts at the seedling stage, 517 DEGs were identified under moderate drought stress, and 683 DEGs were identified under severe drought stress. A total of 137 genes were co-upregulated under both conditions (**Figure 3C**).

**Figure 4:**
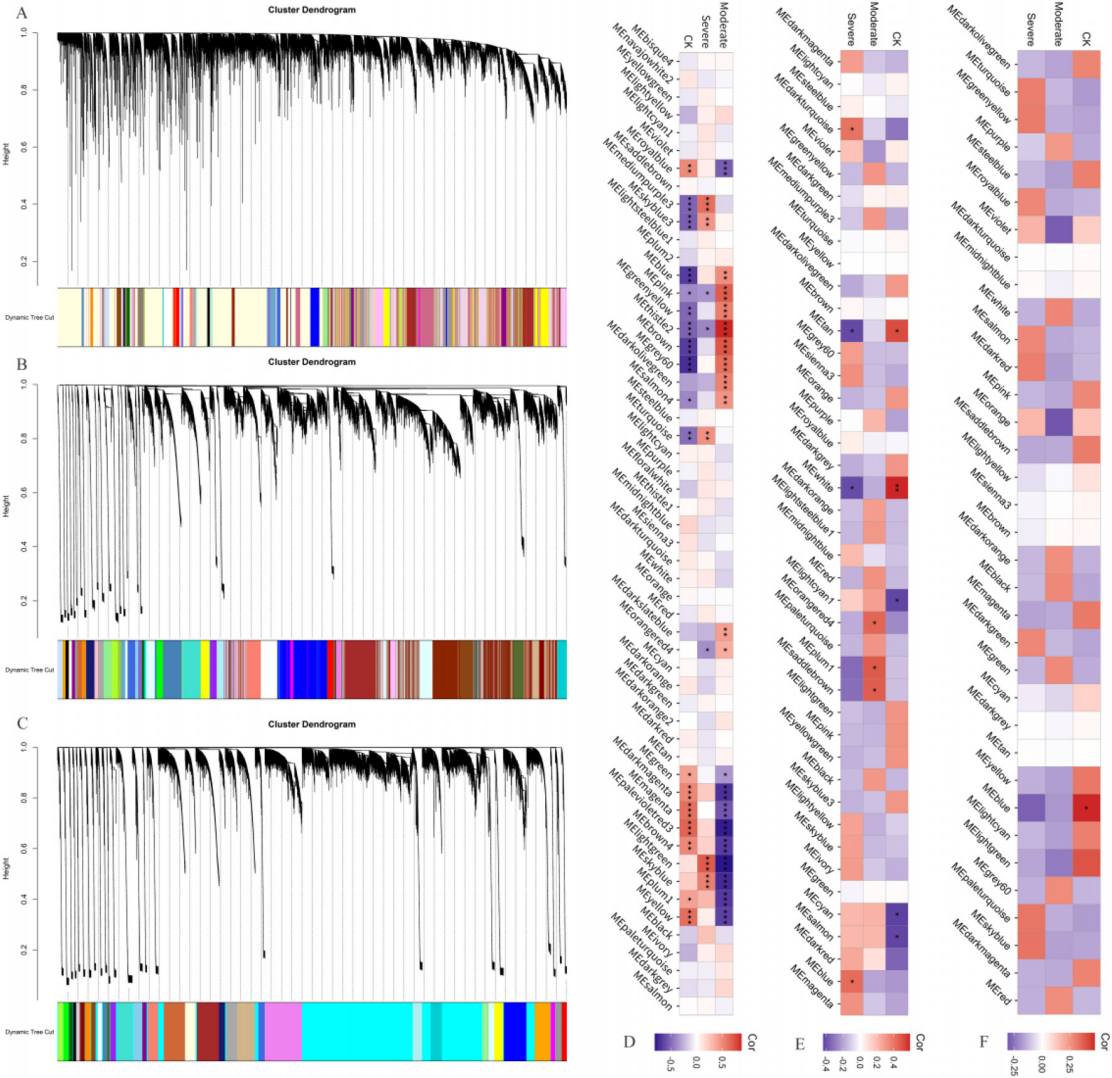
Weighted gene co-expression network analysis. **(A)** Dynamic cutting clustering diagram at germination stage; **(B)** Dynamic cutting clustering diagram of underground at seedling stage; **(C)** Dynamic cutting clustering diagram of aboveground at seedling stage; **(D)** Correlation between modules and treatments at germination stage; **(E)** Correlation between underground modules and treatments at seedling stage; **(F)** Correlation between aboveground modules and treatments at seedling stage. *: P ≤ 0.05; **: P ≤ 0.01; ***: P ≤ 0.001.

To further identify the genes associated with drought tolerance in *B. juncea*, a WGCNA analysis was performed using the FPKM values of the genes. It was found that all genes in the germination stage were divided into 54 modules (**Figure 4A**). Of these, 10 modules (Meblue, Mepink, Megreenyellow, MEthistle2, Mebrown, MEgrey60, Medarkolivegreen, MEsalmon4, Medarkslateblue, MEorangered4) were significantly positively correlated with moderate drought stress (**Figure 4D**), containing 4156, 739, 598, 65, 3264, 318, 120, 58, 73, and 92 genes, respectively. Five modules (MEmediumpurple3, MEskyblue3, Meturquoise, Melightgreen, Meskyblue) were significantly positively correlated with severe drought stress (**Figure 4D**), containing 90, 97, 5146, 273, and 145 genes, respectively. The aboveground genes at the seedling stage were divided into 35 modules, but no significant correlations were observed under both moderate and severe drought stress (**Figures 4C, F**). The underground genes at the seedling stage were divided into 43 modules (**Figure 4B**), and three modules (MEorangered4, MEplum1, Mesaddlebrown) were significantly positively correlated with moderate drought stress (**Figure 4E**), containing 222, 224, and 353 genes, respectively. Two modules (Medarkturquoise and Meblue) were significantly positively correlated with severe drought stress (**Figure 4E**), containing 480 and 3455 genes, respectively.

### 3.5 GWAS and WGCNA joint analysis of key drought-resistant genes

To screen for key drought tolerance genes in *B. juncea* during the germination and seedling stages, a joint analysis of the SNPs co-detected by GWAS (**Supplementary Table S2**) and the WGCNA significantly correlated modules was performed. Under moderate drought stress during the germination stage, twelve co-expression genes were detected in the Megrey60 module and 11 SNPs, including *A02_83231, B03_27425545*, and *B06_8272007* et al. (**Supplementary Table S5**), of which 3 are related to drought tolerance. KEGG pathway analysis primarily enriched in metabolic pathways, such as plant hormone signaling (ko04075), tryptophan metabolism (ko00380), and the plant *MAPK* signaling pathway (ko04016) (**Supplementary Figure S2A**). Pfam functional annotation revealed that the *BjuA040852* gene encodes an auxin-responsive protein (*SAUR*). The *SAUR* gene is predominantly expressed in the growing hypocotyl and other elongated tissues, suggesting its role in regulating cell elongation (Gil and Green, 1997). The *BjuB039062* gene is involved in the synthesis of *LEA* protein, which has been shown to play a role in drought tolerance across various crops. *BjuB039062* is primarily expressed in plant root crowns, leading to the programmed cell death of peripheral cells, which continuously shed from the roots into the surrounding mucus, thereby halting root system growth (Matsuyama et al., 1999). The *BjuB045128* gene encodes glucosinolate, which regulates glucosinolate content in response to drought stress (**Supplementary Figure S2B**).

Three co-expression genes were detected in the MeDarkslateblue module and three SNPs *(B08_12753256, B06_8272007, B03_24227387*), one of which is related to drought tolerance. KEGG pathway enrichment analysis showed significant enrichment in the glycosphingolipid biosynthesis (ko00603) and galactose metabolism (ko00052) pathways (**Supplementary Figure S3A**). Functional annotation of pfam revealed that the *BjuB020721* gene encodes the *AP2/ERF* transcription factor, which is one of the most common transcription factor families in plants. This family has been shown to play a critical role in the regulation of stress responses to drought, high temperature, and salt (**Supplementary Figure S3B**) (Geng et al., 2023; Zha et al., 2023). Four co-expression genes were identified in the Meorangered4 module and four SNPs (*A01_21383845, B08_12753256, B03_31570752, B07_16002213*), one of which is related to drought tolerance. KEGG enrichment analysis revealed a significant pathway in the phosphatidylinositol signaling system (ko04070). Functional annotation of pfam showed that the *BjuB035910* gene regulates a *WRKY* domain, a family of transcription factors known for their role in plant stress responses (Khoso et al., 2022). Under severe drought stress during the germination stage, five co-expression genes were detected in the MEmediumpurple3 module and five SNPs (*A04_2036600, B01_22125408, B01_26022757, B04_7089033, B03_27425545*), two of which are related to drought tolerance. KEGG enrichment analysis highlighted pathways involved in purine metabolism, microbial metabolism in different environments, and plant hormone signal transduction (**Supplementary Figure S4A**). Notably, the *BjuA014514* gene is involved in regulating auxin-responsive proteins (*SAUR*), while the *BjuB027858* gene encodes the *FLZ* zinc finger domain. *FLZ* domain proteins are known to regulate both biotic and abiotic stresses in plants (Jamsheer K and Laxmi, 2015).

Eleven co-expression genes were detected between the Medarkturquoise module and 11 SNPs, including *A03_19182845, B01_3121493*, and *B02_40426592* et al, under moderate drought stress in the underground parts of seedlings (**Supplementary Table S6**). This includes *BjuB035910*, which was also detected under moderate drought stress at the germination stage. KEGG pathway enrichment analysis revealed significant involvement in indole alkaloid biosynthesis, tryptophan metabolism, betaine biosynthesis, and secondary metabolite biosynthesis (**Supplementary Figure S4B**). Under severe drought stress, 109 genes were co-expression with 67 SNPs, including *A06_1565126, A07_11392046*, and *A01_21383845* et al, and four genes were associated with drought tolerance. The KEGG enrichment analysis highlighted key metabolic pathways such as plant hormone signal transduction, material transport, and stress responses. The homologous gene of *BjuB022264* encodes a novel transcriptional regulator in *Arabidopsis thaliana*, which binds specifically to the promoter of *THAS1* (thalianol synthase 1), interacts with ceramides, and is involved in drought and salt tolerance. The *BjuB022292* gene regulates *GATA* transcription factors, which are known to play a role in plant growth, drought resistance, and secondary metabolism. *BjuB022282* encodes the large ribosomal subunit protein *uL29c*, and plastid ribosomal proteins (*PRP*s) are closely linked to the response and adaptation of higher plants to abiotic stresses. Changes in environmental conditions affect plastid gene expression, enabling plants to adapt to internal and external changes. *BjuB022235* participates in various physiological processes, such as plant stress defense and cell wall modification, through Cytochrome *b561* and the *DOMON* domain. No significant correlation modules were detected in the aboveground WGCNA at the seedling stage (**Figure 4F**), and thus no further correlation analysis was conducted.

**Figure 5:**
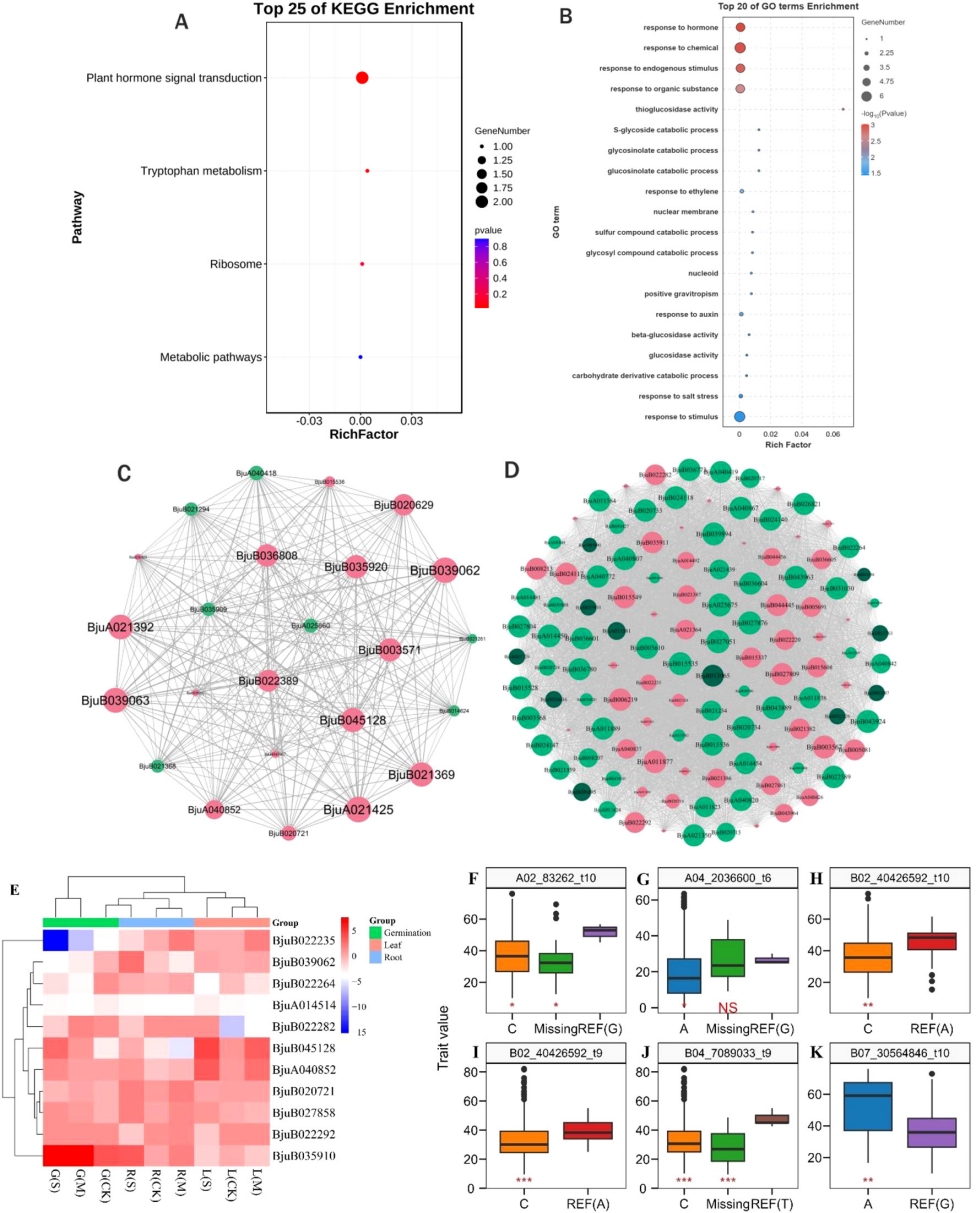
Functional analysis of drought tolerance-related genes and association of SNP alleles with phenotype. **(A)** KEGG enrichment analysis of 8 candidate genes; **(B)** GO annotation analysis of 8 candidate genes; **(C)** Gene co-expression network of Megrey60, MeDarkslateblue, Meorangered4 and MEmediumpurple3 modules at germination stage; **(D)** Gene co-expression network of Medarkturquoise and Meblue modules at seedling stage; **(E)** Expression heat map of drought tolerance-related genes, G(S) indicates severe drought stress at the germination stage,G(M) indicates moderate drought stress at the germination stage,G(CK) indicates control at the germination stage,R(S) indicates severe drought stress at the germination stage, R(S) indicates roots at seedling stage under severe drought stress, R(M) indicates roots at seedling stage under moderate drought stress, R(CK) indicates roots at seedling stage under control, L(S) indicates leafs at seedling stage under severe drought stress, L(M) indicates leafs at seedling stage under moderate drought stress, L(CK) indicates leafs at seedling stage under control. **(F-K)** Association analysis of SNP alleles with phenotypes, t6 indicates germination rate, t9 indicates aboveground fresh weight, t10 indicates underground fresh weight, REF, reference; Missing, missing alleles. NS: P > 0.05; *: P ≤ 0.05; **: P ≤ 0.01; ***: P ≤ 0.001.

Taken together, 11 drought tolerance-related genes were detected through association analysis of SNPs co-detected by WGCNA significant modules and GWAS in the underground part of the seedling stage (**Table 2**). Among them, the *BjuB035910* gene was detected both in the germination stage and in the underground part of the seedling stage. These 11 genes were primarily enriched in plant hormone signal transduction, ribosome, and tryptophan metabolism pathways (**Figure 5A**) and regulated drought stress responses through glucosinolate activity, auxin-responsive proteins, LEA proteins, *GATA, AP2/ERF*, and *WRKY* transcription factors (**Figure 5B**). The gene co-expression network visualization of the candidate gene modules revealed that the connectivity of genes like *BjuA040852, BjuB022264, BjuB022282, BjuB022292, BjuB039062*, and *BjuB020721* was high (**Figures 5C, D**), suggesting these genes may be key nodes in drought tolerance in *B. juncea*. Comparison of gene expression between the drought-tolerant and sensitive groups showed that the expression of the *BjuB022235* gene was significantly down-regulated during germination (log_2_FC = -1.47 to -15.19), with greater down-regulation under more severe drought stress (**Figure 5E**). Conversely, the expression of the *BjuB035910* and *BjuB039062* genes was up-regulated and down-regulated during germination (log_2_FC = 6.21, -2.19), respectively. The expression of the *BjuA014514* gene was down-regulated at both the germination and seedling stages (log_2_FC = -2.65 to -3.87), while the expression of other candidate genes varied across different materials, indicating that the drought tolerance mechanism of *B. juncea* is complex.

**Table 2:** Drought tolerance-related genes detected by WGCNA and GWAS.

Gene_ID	Module	SNP_ID	Chr	SNP_start	SNP_end	Gene_start	Gene_end
BjuA040852	grey60	A02_83262	A02	316738	483262	573108	573440
BjuB039062	grey60	B02_40426592	B02	40026592	40826592	40398825	40400213
BjuB045128	grey60	B07_30564846	B07	30164846	30964846	30353698	30356494
BjuB020721	darkslateblue	B06_8272007	B06	7872007	8672007	8588363	8589031
BjuB035910	orangered4	B03_31570752	B03	31170752	31970752	31916847	31917193
BjuA014514	mediumpurple3	A04_2036600	A04	1636600	2436600	2101643	2102152
BjuB027858	mediumpurple3	B04_7089033	B04	6689033	7489033	7119656	7120336
BjuB022264	blue	B06_24087132	B06	23687132	24487132	23913152	23919228
BjuB022292	blue	B06_24082156	B06	23682156	24482156	24083624	24084788
BjuB022282	blue	B06_24087132	B06	23687132	24487132	24030406	24031368
BjuB022235	blue	B06_24085222	B06	23685222	24485222	23686061	23688163

In order to further analyze the relationship between SNP allele types and phenotypic changes, the SNP loci of 11 drought-tolerance-related genes were associated with germination rate, aboveground and underground fresh weight under moderate and severe drought stress. Among these loci, three SNPs (*A02_83262*, *B02_40426592*, and *B07_30564846*) were found to be significantly correlated with underground fresh weight. The SNP *A02_83262* contains 3 allele types: G in the reference genome (Braju_tum_V1.5), with 2 materials carrying this allele, 174 materials carrying allele type C, and 17 materials missing this SNP locus. For *B02_40426592*, there are 2 allele types: A in the reference genome, with 10 materials carrying this allele and 184 materials carrying allele type C. Regarding *B07_30564846*, it contains 2 allele types: G in the reference genome, with 185 materials carrying this allele and 8 materials carrying allele type A. Comparing the underground fresh weight of materials carrying different alleles, it was observed that the G allele at the *A02_83262* locus, the A allele at the *B02_40426592* locus, and the A allele at the *B07_30564846* locus significantly increased underground fresh weight, indicating better drought tolerance (**Figures 5F, H, K**). Moreover, the *B02_40426592* and *B04_7089033* loci were significantly correlated with aboveground fresh weight. For *B02_40426592*, there are 2 allele types: A in the reference genome, with 12 materials carrying this allele and 181 materials carrying allele type C. As for *B04_7089033*, it contains 3 allele types in the reference genome, with 4 materials carrying this allele, 177 materials carrying allele type C, and 12 materials missing the SNP. Comparing the aboveground fresh weight of materials carrying different alleles, it was found that materials carrying the A allele at the *B02_40426592* locus and the T allele at the *B04_7089033* locus exhibited significantly increased fresh weight, indicating better drought tolerance (**Figures 5I, J**). The *A04_2036600* locus was significantly correlated with germination rate, containing 3 allele types: G in the reference genome, with 3 materials carrying this allele, 184 materials carrying allele type A, and 6 materials missing this SNP locus. Comparing the germination rate of materials carrying different alleles, it was evident that materials carrying G alleles at the *A04_2036600* locus showed a significant increase in germination rate, indicating better drought tolerance (**Figure 5G**).

### 3.6 Regional analysis of B06 chromosome 23.955-24.089Mb

Selective sweep analysis during the germination stage identified two regions on chromosome B06 (8170361–8357420kb, 23686061–24177367kb). WGCNA analysis of the underground at the seedling stage revealed that four drought-related genes in the blue module were also located within the B06 chromosome region of 23686061–24084788 kb (**Table 2**), and all of them were detected in the GWAS under moderate drought stress treatment during the germination stage. It is hypothesized that this region may be an important locus affecting drought tolerance in *B. juncea*.

**Figure 6:**
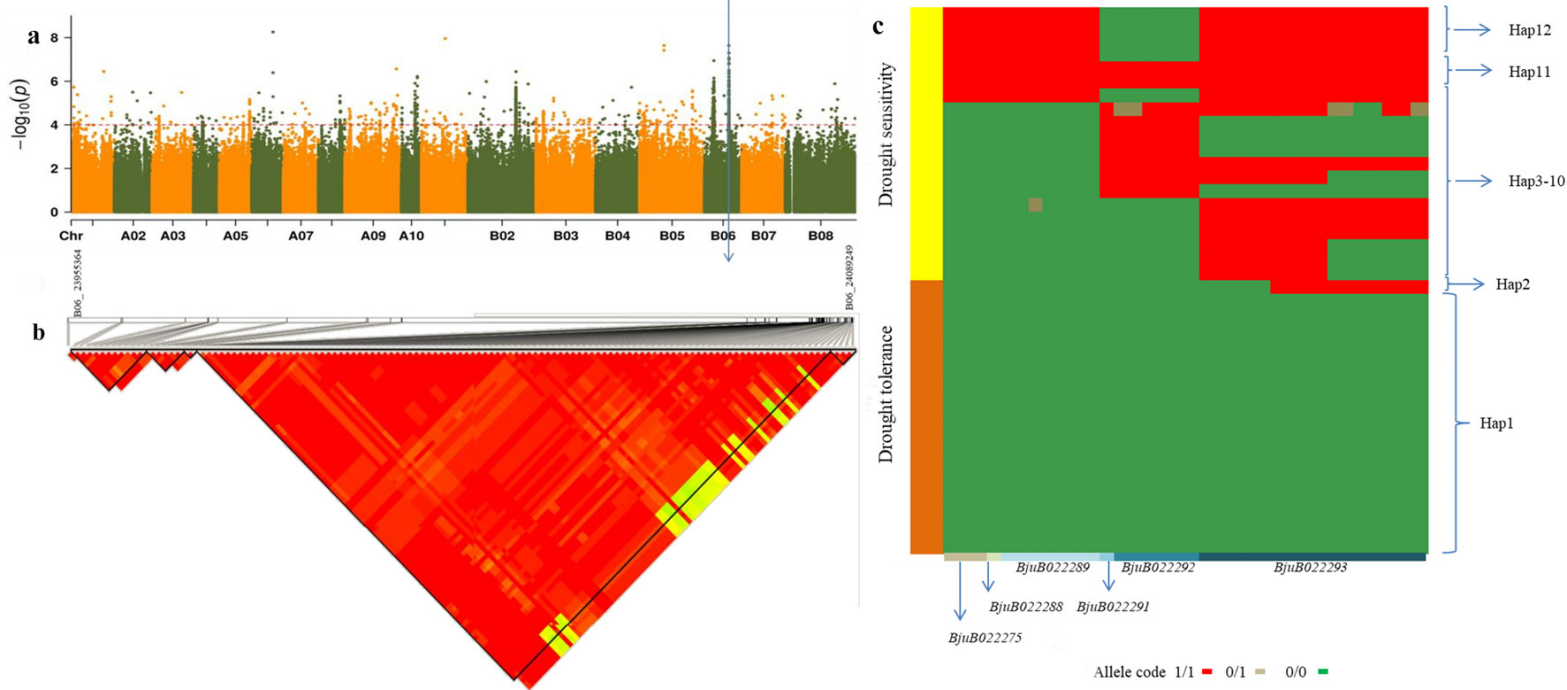
The SNPs and haplotypes responsible for relative germination rate under drought stress during germination period in the region from 23.955-24.089Mb on B06 chromosome. **(a)** Local Manhattan plot showing the surrounding the candidate region. The position marked by the blue arrow was the significantly correlated SNP screened out. **(b)** LD heat map of the region of the candidate region. **(c)** The haplotype of the chromosome B06 region between 23.955 Mb and 24.089Mb. *BjuB022275, BjuB022288, BjuB022289, BjuB022291, BjuB022292*, and *BjuB022293* are 6 genes that contain 33 SNPs. 1/1, 0/1, and 0/0 are allele types. 0/0 indicates that it is identical to the reference genome, 0/1 indicates partial divergence from the reference genome, and 1/1 indicates complete divergence from the reference genome. Hap1-Hap12 are 12 haplotypes.

SNP scanning in this region revealed that the SNPs in the interval of B06 chromosome (23955364–2408949 kb) were in high linkage disequilibrium. A total of 125 SNPs were found to be associated with seed germination rate (**Figure 6a**). Among these, 33 loci could roughly distinguish 193 materials as drought-tolerant or drought-sensitive at the germination stage (**Supplementary Table S7**). Additionally, 12 haplotypes were identified (**Figure 6b**), with Hap1 exclusively found in extremely drought-tolerant materials. In fact, 95% of the extremely drought-tolerant materials were Hap1, while one material was Hap2. Hap3 to Hap12 were primarily associated with drought-sensitive materials. Of the 33 loci mentioned above, 26 are located in the coding region of genes, including 18 missense or stop codon mutations and 8 synonymous mutations (**Figure 6c**; **Supplementary Table S7**). Fourteen of the 18 non-synonymous mutation sites are located in the exon region.

## 4 Discussion

Seed germination is a crucial stage in the life cycle of plants. Adverse environmental factors, such as drought and low temperature, can stunt seed development and inhibit seedling emergence (Hussain et al., 2018). In this study, the germination rate of *B. juncea* (193 materials) was significantly reduced under drought stress. The negative impact was more pronounced in drought-sensitive materials than in drought-tolerant ones. Drought stress lowers the water potential difference between the internal and external environments of seeds, reducing the rate and total amount of water entering the seed coat. It also slows the hydrolysis of dry matter in the cotyledons and impedes the transport of nutrients to the developing mesoembryo, thus hindering seed germination and growth (Boureima et al., 2011; Ivanov et al., 2019). Additionally, drought stress caused both aboveground and underground parts of seedlings to shorten, with the aboveground part showing a greater degree of shortening than the underground part, leading to an increased root-to-shoot ratio. Studies have shown that the shortening of the underground part under drought stress is related to a decrease in cell division and number (Ashraf et al., 2013; Batool et al., 2022). Moreover, drought stress induces changes in the levels of cytokinin (CTK), auxin *(*IAA), and gibberellin (GA) in rape roots, which modulate root structure changes, enhancing drought resistance and promoting water and mineral uptake (Mi et al., 2023). In this study, we identified that the *BjuA040852* and *BjuA14* genes encode auxin-responsive proteins (*SAUR*), which are mainly expressed in the growing hypocotyl or other elongated tissues, regulating hypocotyl elongation and development (Gil and Green, 1997). The *BjuB039062* gene, primarily expressed in the root crown, causes programmed cell death in the peripheral cells of the root crown, halting root growth (Matsuyama et al., 1999). This may contribute to the shortening of the underground parts under drought stress. The 193 materials were classified into 24 drought-tolerant, 139 moderately drought-tolerant, and 30 drought-sensitive materials, indicating that most materials exhibited moderate drought tolerance. However, there are still few varieties with strong drought tolerance in production, which are insufficient to cope with climate change, environmental shifts, and extreme weather events.

Genome-wide association studies (GWAS) are widely used in crops such as rapeseed, rice, and wheat as an effective method for detecting QTLs, providing higher resolution and more cost-effective characteristics for QTL mapping or gene discovery compared to traditional parental linkage mapping (Liu et al., 2016; Yuan et al., 2020b). In this study, the results of GWAS analysis revealed that six QTLs related to drought tolerance at the germination sta*0145*ge were distributed on chromosomes A04, A07, B03, and B06. Of these, five were significantly associated with RG under moderate drought stress, while one was significantly associated with RG under severe drought stress. A total of 21 QTLs related to drought tolerance were detected at the seedling stage, mainly distributed on chromosomes A01, A02, A03, B01, B02, B04, B06, and B08. Among these, eight were significantly correlated with seedling RWA, and 13 were significantly correlated with seedling RWR. All 27 QTLs were detected in two or more environments simultaneously, and it is noteworthy that one QTL associated with severe drought stress RG on A04 was detected in all three environments, indicating that these QTLs can be stably inherited across different environments.

In this study, 11 drought tolerance genes in *B. juncea* were identified, which are involved in various pathways such as plant hormone signal transduction, ribosomal processes, and tryptophan metabolism. Among them, the *BjuA040852* and *BjuA014514* genes encode the auxin-responsive protein *SAUR* (small auxin upregulated RNA), which is predominantly expressed in growing hypocotyls and other elongated tissues. The SAUR gene family is the largest family of early auxin response genes in higher plants, and plays a key role in regulating a variety of biological processes, including auxin synthesis and trafficking, apical development, leaf and root growth, and senescence (Wang et al., 2020b; Li et al., 2021). Additionally, the *SAUR* gene dynamically regulates plant growth in response to both internal and external environmental stimuli (Stortenbeker and Bemer, 2019). Li et al. identified 253 alleles of *GmSAUR* with excellent drought tolerance in wild soybean through haplotype analysis. RNA-Seq data revealed that six *GmSAUR* genes were induced under dehydration stress, and a haplotype potentially associated with drought tolerance was identified in the *GmSAUR299* promoter (Li et al., 2022). Similarly, Liu et al. (2022) conducted a genome-wide analysis and identified 162 *SAUR* genes in the peanut genome, which are widely involved in the response to abiotic stresses (Liu et al., 2022). Functional analysis revealed that *AhSAUR3* negatively regulates drought tolerance in peanuts. Moreover, auxin-regulated transcription factor *AP2/ERF* has been shown to play a crucial role in response to abiotic stress. The *ERF* and *RAV* transcription factors are primarily involved in the stress response (Xie et al., 2019). Salt and PEG treatments rapidly induce the expression of *ERF* and *RAV* TFs, thereby enhancing the rice plant’s stress adaptability (Wang et al., 2020b). In this study, it was found that the *BjuB020721* gene improves the response of *B. juncea* to drought stress by regulating the *AP2/ERF* transcription factor. Regarding the synergistic effect of *SAUR* genes and *ARF* transcription factors in mediating plant hormone signaling and drought tolerance, Meng et al. proposed that the *SAUR* gene down-regulates the expression of auxin in the roots, regulating the growth rates of both the roots and plant height to alleviate water shortage (Meng, 2018). Additionally, auxin-responsive factors (*ARFs*) may bind to the transcriptional repressor Aux/IAA and inhibit auxin synthesis, thereby reducing the accumulation of plant biomass to further alleviate water scarcity (Ma et al., 2023). the *BjuB035910, BjuB022292, BjuB022264*, and *BjuB027858* genes regulate the *WRKY* domain, *GATA* transcription factor, transcription factor binding specifically to the *THAS1* promoter (thalianol synthase 1), and the *FLZ* zinc finger domain, respectively, in response to drought stress in mustard rapeseed. The *WRKY* transcription factor (TF) gene family plays an important role in the transcriptional regulation of plant stress responses. The Arabidopsis *WRKY57* transcription factor may confer drought tolerance in transgenic rice (*Oryza sativa*) plants. Overexpression of *AtWRKY57* in rice enhances tolerance to drought, salinity, and polyvinyl alcohol (*PVA*)-induced stress (Jiang et al., 2016). Additionally, transferring the *SbWRKY30* gene from sorghum into Arabidopsis thaliana led to high expression of the *SbRD19* gene, and overexpression of *SbRD19* improved drought tolerance in Arabidopsis compared to wild-type (WT) plants. This suggests that *SbWRKY30* acts as a positive regulator in response to drought stress (Yang et al., 2020). Under drought stress, *MaWRKY80* promotes stomatal movement and water retention in *Arabidopsis* leaves by regulating the transcription of 9-cis-epoxycarotenoid dioxygenase (NCED) and abscisic acid (ABA) biosynthesis (Liu et al., 2020). Overexpression of *GhWRKY68* in *Nepenthes bicalcarata* can respond to drought and salt stress by modulating ABA signaling and regulating cellular reactive oxygen species (ROS) (He et al., 2019). *GATA* transcription factors recognize *GATA* motifs and specifically bind to promoter sequences (A/T)*GATA*(A/G) (Du et al., 2022). Most *GATA* transcription factors contain one or two highly conserved type IV zinc finger protein domains. *GATA* genes have been identified in various plant species and are mainly involved in processes such as seed germination, chloroplast development, plant flowering time control, and stress responses (Hannon et al., 1991). In poplars, *PdGNC1* mediates the production of NO and H_2_O_2_, reducing stomatal closure by binding to the promoter of *PdHKX1* under drought stress (Shen et al., 2021). FCS-like zinc finger family proteins (*FLZs*) are plant-specific scaffolds for the *SnRK1* complex, regulating various aspects of plant growth and stress responses. Salt stress-induced ectopic expression of the wheat *FLZ* gene *TaSRHP* in *Arabidopsis thaliana* enhances tolerance to salt and drought stress (Chen et al., 2021). Both *AtFLZ6* and *AtFLZ10* knockdown mutants of Arabidopsis show increased sensitivity to root elongation inhibition by abscisic acid (ABA) and reduced tolerance to osmotic stress (Hou et al., 2013; Jamsheer K et al., 2019). The *BjuB022282* gene encodes a subunit of plastid ribosomal proteins (*PRPs*) (*uL29c*), and a close association between *PRPs* and tolerance to adverse environmental conditions has been identified. *PRPs* are involved in both developmental processes and responses to abiotic stresses (Robles and Quesada, 2022). Liu et al. overexpressed *RPL44* in yeast and tobacco, which resulted in increased tolerance to salt and drought stress (Liu et al., 2014). The *BjuB022235* gene is involved in plant stress defense and cell wall modification through Cytochrome *b561* and the *DOMON* domain. It was found that *CLb561A* mRNA and protein levels were significantly elevated in watermelon leaves under high light intensity and drought stress. The excess energy absorbed by chloroplasts was transported to the apoplast, where it was safely dissipated through the synergistic action of cytochrome *b561* and *AO*. This forms a self-protection mechanism against photodamage and confers good tolerance to high light intensity and severe drought (Nanasato et al., 2005; Muhammad Ashraf et al., 2013).The expression of the *LEA* genes *PMA80* and *PMA1959* in wheat has been shown to increase the dehydration tolerance of transgenic rice. Similarly, overexpression of the *OsEm1* gene encoding the *LEA* protein has also enhanced the drought tolerance of rice (Hu et al., 2016; Yu et al., 2016). Studies have shown that *LEA* proteins are a large group of hydrophilic proteins with various functions, including protecting cell structures from dehydration-induced damage, ion isolation, and the folding of denatured proteins (Serrano and Montesinos, 2003; Grelet et al., 2005). Additionally, they can act as chaperone proteins to protect cells from membrane damage. The second group of the *LEA* protein family, also known as dehydrins (DHN), are highly hydrophilic proteins. As molecular chaperones, dehydrins provide dual functions by offering cell protection and repairing damage in cells exposed to stress, thereby providing stability to biomolecules and proteins (Halder et al., 2016). These functions may help protect plants from oxidative damage. The *BjuB045128* gene encodes glucosinolate, which regulates glucosinolate content in response to drought stress. Glucosinolates and their degradation products play a key role in plant responses to environmental stresses, particularly in processes such as heat tolerance, water transport, and transcriptional reprogramming (Brader et al., 2006; Kissen et al., 2016). Exogenous application of glucosinolate degradation products (e.g., isothiocyanates, nitriles, and thiocyanates) has been shown to induce stomatal closure, thereby reducing water loss (Hossain et al., 2013), while application of glucosinolate derivatives significantly reduces stomatal pore size (Eom et al., 2018). Plant response to drought stress is a complex regulatory network, when plants are exposed to water-deficient conditions, the *SAUR* gene and *ARF* transcription factors mediate plant hormone signaling to regulate root and shoot growth, thereby increasing water absorption. *WRKY* and *GATA* transcription factors reduce stomatal movement to minimize water evaporation, while the *b561* protein forms a photoprotective mechanism to prevent light damage. *LEA* hydrophilic proteins, acting as chaperone proteins, protect cells from membrane damage and enhance protein stability, forming a regulatory network that helps plants cope with drought stress. In this study, several drought tolerance-related genes were identified through GWAS and WGCNA, and the functions of these genes and their roles in drought response pathways were analyzed, providing new insights into drought tolerance in *B. juncea*. However, the precise functions of these genes and their complex regulatory networks still need further exploration. Combined with production needs and the global agro-climate trends, more effective varieties and breeding strategies that are tolerant to multiple stresses should be developed. This will lay a solid foundation for improving canola productivity and coping with global environmental changes.

## Data Availability

The datasets presented in this study can be found in online repositories. The names of the repository/repositories and accession number(s) can be found in the article/**Supplementary Material**.
