# Supplementary material for: GWAS and WGCNA analysis uncover candidate genes associated with drought in Brassica juncea L
Source: Front Plant Sci. 2025 Apr 4;16:1551804. doi: 10.3389/fpls.2025.1551804 (PMC12007043; doi:10.3389/fpls.2025.1551804)
Supplement: Supplementary file 2 [file SupplementaryFile1.pdf]

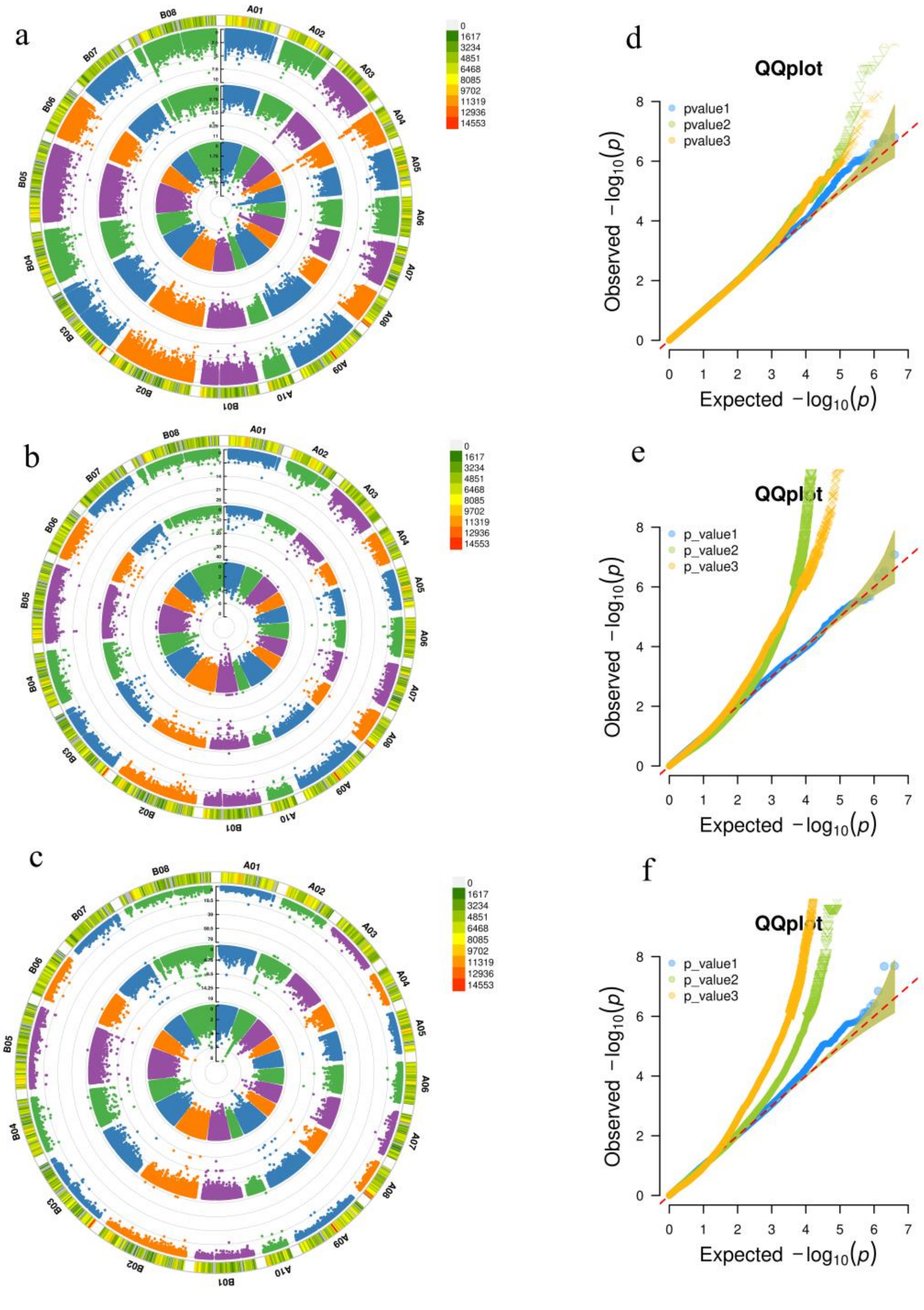

Fig.S1 Genome-wide association analysis of Manhattan plot and QQ plot.

Note: a-c are the Manhattan plots of the relative germination rate, the aboveground and underground fresh weight at the seedling stage between 3 years (2019, 2020 and 2021) under severe drought stress. **d-f** are the QQ plots of relative germination rate, abovegroun and underground fresh weight at seedling stage in 3 years under severe drought stress.

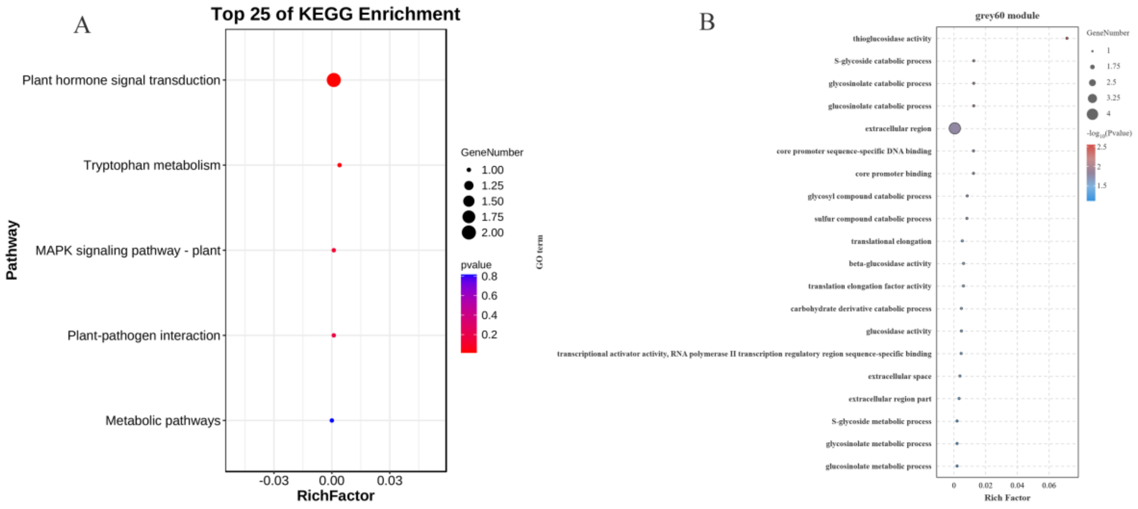

Fig.S2 KEGG and GO analysis of co-expressed genes in the Megrey60 module

Note: **A** is the KEGG enrichment analysis of co-expressed genes in the Megrey60 module; **B** is the GO enrichment analysis of co-expressed genes in the Megrey60 module.

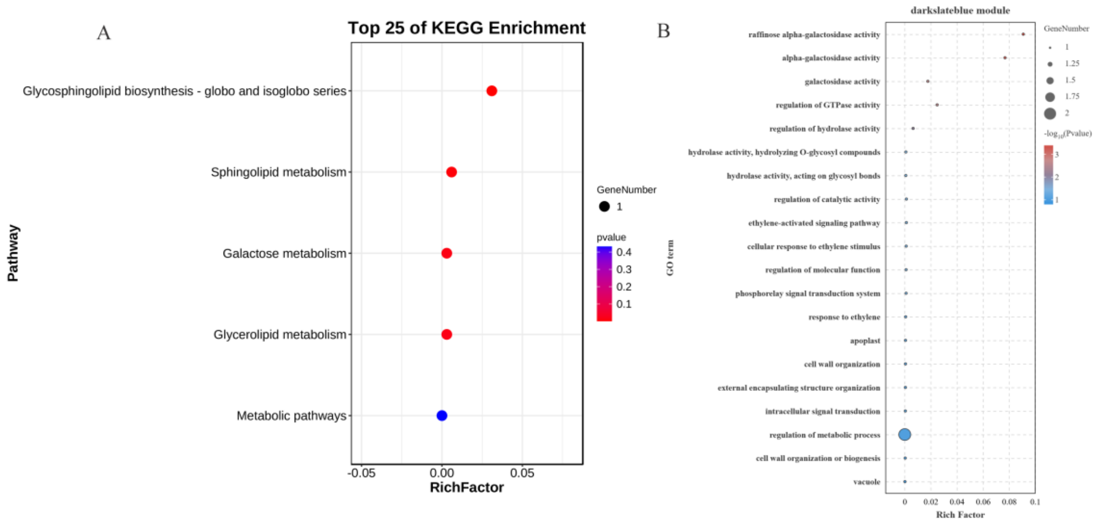

Fig.S3 KEGG and GO analysis of co-expressed genes in the MeDarkslateblue module

Note: **A** is the KEGG enrichment analysis of co-expressed genes in the MeDarkslateblue module; **B** is the GO enrichment analysis of co-expressed genes in the MeDarkslateblue module.

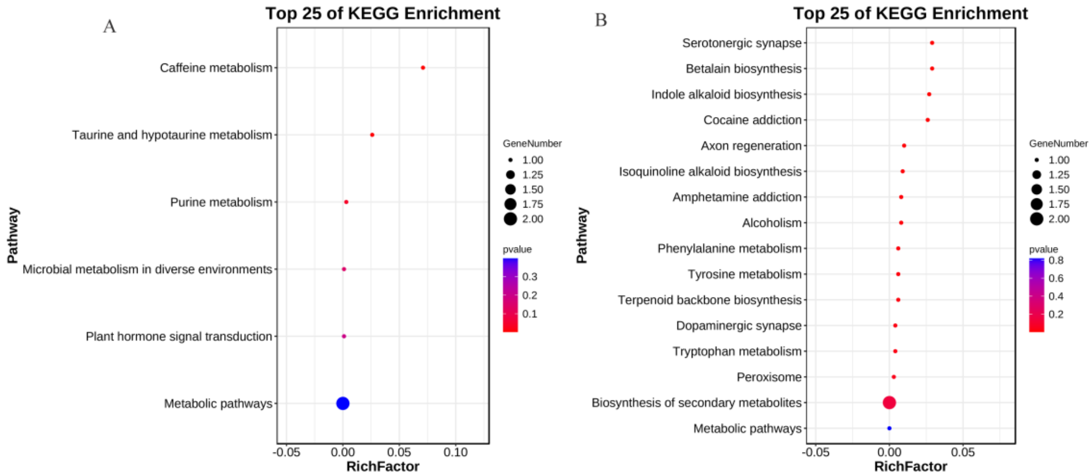

Fig.S4 KEGG analysis of co-expressed genes

Note: **A** is the MEmediumpurple3 module;**B** is the Medarkturquoise module
